# Obsessive-Compulsive Disorder and Autism Spectrum Disorders: Longitudinal and Offspring Risk

**DOI:** 10.1371/journal.pone.0141703

**Published:** 2015-11-11

**Authors:** Sandra M Meier, Liselotte Petersen, Diana E Schendel, Manuel Mattheisen, Preben B Mortensen, Ole Mors

**Affiliations:** 1 National Centre for Register-Based Research, Aarhus University, Aarhus C, DK; 2 Department of Public Health, Section of Epidemiology, Aarhus University, Aarhus C, DK; 3 Department of Biomedicine, Aarhus University, Aarhus C, DK; 4 Research Department P, Aarhus University Hospital, Risskov, DK; 5 The Lundbeck Foundation Initiative for Integrative Psychiatric Research, iPSYCH, DK; Chiba University Center for Forensic Mental Health, JAPAN

## Abstract

**Background:**

Despite substantial similarities and overlaps in the pathophysiology of obsessive-compulsive disorders (OCD) and autism spectrum disorders, little is known about the clinical and etiologic cohesion of these two disorders. We therefore aimed to determine the patterns of comorbidity, longitudinal risks, and shared familial risks between these disorders.

**Methods:**

In a prospective study design we explored the effect of a prior diagnosis of OCD in patients and parents on the susceptibility to autism spectrum disorders and vice versa. Analyses were adjusted for sex, age, calendar year, parental age and place at residence at time of birth. As measures of relative risk incidence rate ratios (IRR) and accompanying 95% confidence intervals (CIs) were employed.

**Results:**

The risk of a comorbid diagnosis of OCD in individuals with autism spectrum disorder and aggregation of autism spectrum disorders in offspring of parents with OCD were increased. Individuals first diagnosed with autism spectrum disorders had a 2-fold higher risk of a later diagnosis of OCD (IRR = 2.18, 95% CI = 1.91–2.48), whereas individuals diagnosed with OCD displayed a nearly 4-fold higher risk to be diagnosed with autism spectrum disorders (IRR = 3.91, 95% CI = 3.46–4.40) later in life. The observed associations were somewhat stronger for less severe types of autism spectrum disorders without a comorbid diagnosis of mental disabilities.

**Conclusions:**

The high comorbidity, sequential risk, and shared familial risks between OCD and autism spectrum disorders are suggestive of partially shared etiological mechanisms. The results have implications for current gene-searching efforts and for clinical practice.

## Introduction

Autism spectrum disorders constitute a group of neuro-developmental disorders characterized by severe impairments in social interaction and communication, often accompanied by restricted, repetitive or stereotyped interests and behaviors. Consistent increments in the observed prevalence of autism spectrum disorders are considered a pressing challenge to the global public health system of the 21^st^ century [1]. Currently up to 2% of children worldwide are estimated to be diagnosed with an autism spectrum disorder [1, 2]. There is considerable evidence that patients with autism spectrum disorders are at an increased risk of comorbid anxiety disorders [3–6]. Many patients with autism spectrum disorders display a lack of fear to real dangers, yet may exhibit excessive fearfulness in response to harmless objects. A recent systematic review revealed that approximately 40% of patients with autism spectrum disorders are assigned at least one comorbid diagnosis of anxiety, the most frequent being specific phobia (30%) followed by obsessive-compulsive disorder (OCD; 17%) [7]. Given that the prevalence of OCD in the general population is estimated around 1.6% [8], it appears that OCD is more prevalent among patients with autism spectrum disorders than in the general population.

Comorbidity of OCD and autism spectrum disorder is further reflected in common features of treatment and brain pathophysiology. Antidepressants, especially selective serotonin reuptake inhibitors, constitute the pharmacological treatment of choice for most patients with OCD [9]. Not many medications are truly effective in the treatment of autism spectrum disorders [10], although recent studies indicated that antidepressants might be of value in treatment of autism [11–13]. Furthermore, similar brain structure abnormalities were found in patients with OCD and autism spectrum disorders [14]. In contrast to patients with other anxiety disorders patients with OCD displayed increased gray matter volumes in the caudate nuclei [15, 16]. Structural changes in this limbic area are also described in autism spectrum disorders [17, 18]. Such similarities and overlaps in putative pathophysiology are quite rare and apply to only a fraction of clinical samples.

Exploring to what degree OCD and autism spectrum disorders recur in families and are comorbid conditions might provide crucial insights into etiology and treatment of the two disorders. We therefore examined the comorbidity patterns of OCD and autism spectrum disorders and sequential risks of these disorders. Finally, we investigated the risks of autism spectrum disorders in offspring of individuals with OCD, and vice versa.

## Materials and Methods

### Registers

The Danish Civil Registration System was established in 1968 and provides information on demographic data such as gender, date of birth, vital status (continuously updated) of all persons living in Denmark [19]. All residents of Denmark are assigned a unique personal identification number within the registration system, which can be used for accurate linkage with other national registries.

The Danish Psychiatric Central Register has been computerized since 1969 and it currently comprises data on roughly 855,000 persons and 3.91 million contacts, comprehensively mapping the psychiatric contacts of the entire population of Denmark [20]. The register stores data on all admissions to Danish psychiatric inpatient facilities, and including all outpatient contacts to psychiatric services since 1995. As treatment in Danish hospitals is free of charge for all residents, it can be assumed that psychiatric admissions represented in the Danish Psychiatric Central Register are unbiased. The Danish National Hospital Registry represents all inpatients treatments at non-psychiatric facilities since 1977, including outpatient and emergency room contacts since 1995 [21, 22]. Clinical diagnoses were assigned according to the Danish modification of the International Classification of Diseases, 8th Revision (ICD-8) from 1969 to 1993; since 1994, diagnoses have been assigned on the basis of the ICD-10 Classification of Mental and Behavioral Disorders, diagnostic criteria for research (ICD-10-DCR).

### Study Population

The cohort sample covers all individuals born in Denmark between January 1, 1955, and November 31, 2006. Parents of all individuals in the cohort were required to be known resulting in a total of 3,380,170 cohort members.

### Assessment of Autism Spectrum disorder and Other Mental Illness

For cohort members and their parents, data were extracted for diagnoses of autism spectrum disorders (ICD-8 codes: 299.00 and 299.01; ICD-10 codes: F84.0–F84.12, F84.5-F84.9), OCD (ICD-8 codes: 300.39; ICD-10 codes: F42), anxiety disorders (ICD-10 codes: F40.00-F40.20, F41.00-F41.10, F43.00-F43.10), depression (ICD-8 codes: 296.x9, 298.09, 298.19, 300.49, 301.19; ICD-10 codes: F32.00-F33.99, F34.10-F34.90, F38.00-F39.99), attention deficit hyperactivity disorder (ICD-8 codes: 308.01; ICD-10 codes: F90.x), and mental disabilities (ICD-8 codes: 311.xx-315.xx, ICD-10 code: F70-F79) assigned by adult or child psychiatrists. Additionally, we assessed their psychiatric history, whether they had ever been admitted to a psychiatric hospital or been in outpatient care for a diagnosis of a psychiatric disorder (ICD-8 codes: 290–315; ICD-10 codes: F00–F99). Date of diagnosis was defined as the first contact that led to the diagnosis of interest, irrespective of other previous psychiatric diagnoses in the case history. Parental diagnoses (in either one of the parents) were classed hierarchically as non-mutually exclusive events. Information about parental age and place of residence at time of birth was obtained from the Danish Civil Registration System. For research purposes all personal information from the registers are anonymized. The Danish Data Protection Agency fully approved the study.

### Data Analyses

The data were analyzed using a survival approach. Cohort members were followed from birth or January 1, 1994 (whichever occurred latest) until the onset of the disorder of interest, date of death, date of emigration from Denmark, or December 31, 2012, whichever occurred first. The incidence rate ratio (IRR—a measure of relative risk) was estimated using a log linear Poisson regression model using the GENMOD procedure in SAS, version 9.3 (SAS Institute, Cary, NC, USA). All analyses were adjusted for calendar year, age, maternal and paternal age at birth of child, sex, place of residence at time of birth (as described elsewhere [23]) and the interaction of age with sex. In the analyses assessing sequential risk, we added to the first model, parental psychiatric history, and the occurrence of an OCD diagnosis, and to second model parental psychiatric history, the occurrence of an OCD diagnosis, and the first hospital contact for any other psychiatric disorders of the patient as time dependent variables. Parental effects were estimated using a hierarchical model. Sensitivity analyses were performed for severity, coding (comparing ICD-8 and ICD-10 codes), clinical presentation of OCD, and comorbid mental disabilities in autism spectrum disorders. Patients that require hospitalization are likely to display a more severe type of autism spectrum disorders and OCD. We therefore examined whether both cases with prior in- or outpatients contact were at a higher risk. The classification of autism spectrum disorders and OCD changed overtime; thus we explored the comparability of results based on both ICD-8 and ICD-10 codes or on the newer ICD-10 codes alone. The clinical picture of OCD may be dominated by obsessional thoughts or compulsive acts, accordingly we assessed whether cases with a specific type of OCD were at higher risk to subsequently be diagnosed with autism spectrum disorders. Several studies described the etiology of autism spectrum disorders with and without comorbid mental disabilities to be quite different [24–26]; herein we aimed to determine whether the association with OCD differs for these two forms of autism.

## Results

### Comorbidity of Autism Spectrum Disorders and OCD

Among the 3,380,170 cohort members followed from 1994 to 2012, we observed that 18,184 were diagnosed with an autism spectrum disorder and 11,209 with OCD. This corresponds to a crude incidence rate of 3.2 per 10,000 person-years for autism spectrum disorders and 2.0 for OCD. 739 individuals were diagnosed with autism spectrum disorders and OCD; 281 were first assigned a diagnosis of OCD, 253 a diagnosis of autism spectrum disorders, and 205 were simultaneously diagnosed with OCD and autism spectrum disorders. Individuals with OCD had a 13 times higher risk of having a comorbid autism spectrum diagnosis (6.6%) compared with individuals without OCD (0.5%). For more formation on comorbidities among individuals with OCD or autism spectrum disorders see S1 Table.

### Familial Risks of Autism Spectrum Disorders and OCD

**Fig 1:**
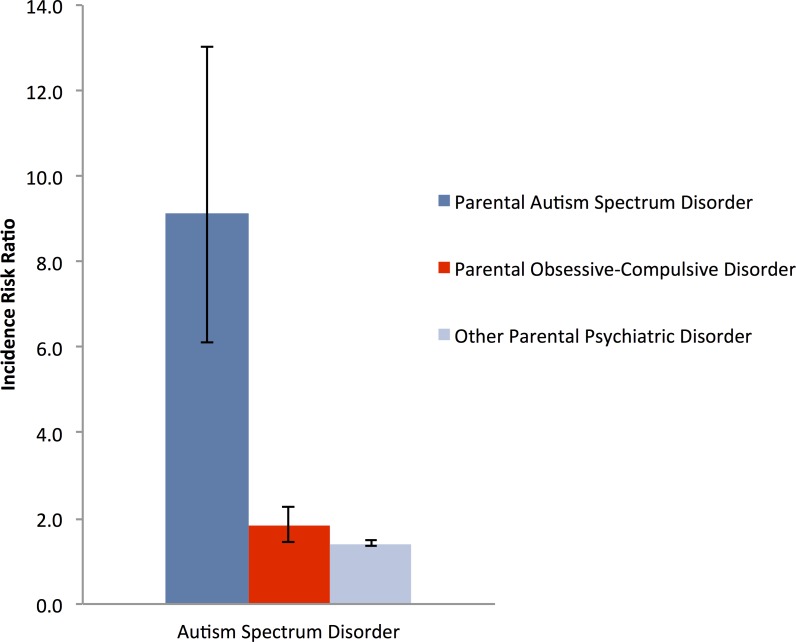
Incidence rate ratios, with 95% CIs (error bars), of Autism Spectrum Disorders in Offspring of Parents with an Obsessive Compulsive Disorder, 1995–2012.

The parents of 86 individuals diagnosed with autism spectrum disorder had been previously diagnosed with OCD. Parental OCD increased the IRR for autism spectrum disorders in their offspring to 1.83 (95% CI = 1.45–2.28); there was no difference in the paternal (IRR = 1.87, 95% CI = 1.21–2.74) or maternal (IRR = 1.74, 95% CI = 1.32–2.25) diagnosis of OCD on the offspring’s risk for autism spectrum disorders. The risk for autism spectrum disorders was somewhat increased after a parental diagnosis of OCD compared to the risk after a parental diagnosis of any psychiatric disorder (IRR = 1.41, 95% CI = 1.36–1.47), but less than the risk after a parental diagnosis of autism spectrum disorders (IRR = 9.13, 95% CI = 6.10–13.02, see Table 1 and Fig 1).

**Table 1:** Incidence Risk Ratio of Specific Diagnoses of Autism Spectrum Disorders in Relation to Individual and Parental Diagnosis of Obsessive-compulsive Disorder (OCD; 1994–2012).

	Prior Diagnosis of OCD in Parents^a^		Prior Diagnosis of OCD in Patients^b^	
Specific Diagnosis of Autism Spectrum Disorder	Incidence Rate Ratio	95% CI	Incidence Rate Ratio	95% CI
Childhood Autism	1.92	1.24–2.82	3.28	2.40–4.35
Atypical Autism	1.63	0.51–3.81	4.40	2.65–6.83
Asperger's Syndrome	2.06	1.38–2.94	5.45	4.54–6.46
Other Pervasive Developmental Disorder	2.05	1.10–3.45	9.39	7.50–11.59
Unspecified Pervasive Developmental Disorder	1.50	0.92–2.28	4.10	3.20–5.15

^a^Maternal or paternal diagnoses were categorized hierarchically as having a history of OCD, autism spectrum disorders or other psychiatric disorders. Estimates of relative risk were adjusted for calendar year, age, maternal and paternal age, sex, place of residence at time of birth and the interaction of age with sex

^b^Estimates of relative risk were adjusted for calendar year, age, maternal and paternal age, sex, parental history of psychiatric illness, first psychiatric hospital contact for any other disorder, place of residence at time of birth and the interaction of age with sex

A parental diagnosis of OCD increased the risk specifically for childhood autism (IRR = 1.92, 95% CI = 1.24–2.82), Asperger’s syndrome (IRR = 2.06, 95% CI = 1.38–2.94), or other pervasive developmental disorder (IRR = 2.05, 95% CI = 1.10–3.45; see Table 1). But only the risk to develop Asperger’s syndrome was significantly higher among offspring of parents with OCD compared to parents with any other psychiatric disorder (IRR = 1.33, 95% CI = 1.25–1.42). The parents of only 4 individuals diagnosed with OCD had previously been diagnosed with autism spectrum disorder. Probably due to this small sample size, we observed a somewhat increased risk for OCD in offspring of parents diagnosed with autism spectrum disorder (IRR = 3.08, 95% CI = 0.96–7.16) that fell just short of significance (see Table 2).

**Table 2:** Incidence Risk Ratio of Obsessive-Compulsive Disorder in Relation to Individual and Parental Diagnoses of Autism Spectrum Disorders (ASD; 1994–2012).

	Risk of Obsessive-Compulsive Disorder	
Diagnosis of Autism Spectrum Disorder	Incidence Rate Ratio	95% CI
Parental Diagnosis of Autism	3.08	0.96–7.16
Individidual Diagnosis of Autism	2.51	2.20–2.84
Individidual Diagnosis of Childhood Autism	1.39	0.98–1.91
Individidual Diagnosis of Atypical Autism	0.63	0.16–1.65
Individidual Diagnosis of Asperger's Syndrome	3.05	2.49–3.69
Individidual Diagnosis of Other Pervasive Developmental Disorder	1.99	1.40–2.71
Individidual Diagnosis of Unspecified Pervasive Developmental Disorder	3.5	2.80–4.32

Estimates of relative risk were adjusted for calendar year, age, maternal and paternal age, sex, parental history of psychiatric illness, first psychiatric hospital contact for any other disorder, place of residence at time of birth and the interaction of age with sex

### Longitudinal Analyses of Autism Spectrum Disorders and OCD

Longitudinal analyses showed that individuals first diagnosed with autism spectrum disorders had a 2-fold higher risk (IRR = 2.18, 95% CI = 1.91–2.48) of receiving a later diagnosis of OCD compared with individuals without autism spectrum disorder during the follow-up period. The independent effect of a prior autism spectrum diagnosis over and above the effect of a psychiatric hospital contact per se was estimated to increase the IRR of OCD to 2.51 (95% CI = 2.20–2.84). Excluding the first year after diagnosis, the effect of a prior autism spectrum diagnosis on the risk to be diagnosed with OCD was relatively stable overtime (see Table 3).

**Table 3:** Incidence Rate Ratio of Obsessive-Compulsive Disorder in Persons with a Diagnosis of Autism Spectrum Disorders (ASD; 1994–2012).

		First Adjustment^a^		Second Adjustment^b^	
Time Since ASD Diagnosis	Cases	Incidence Rate Ratio	95% CI	Incidence Rate Ratio	95% CI
< 1 year	57	3.89	2.96–5.01	3.72	2.83–4.79
1 year	33	2.39	1.66–3.32	2.39	1.66–3.30
2 years	29	2.22	1.51–3.14	2.32	1.57–3.27
3 years	45	1.98	1.45–2.63	2.20	1.62–2.92
5 years	62	1.71	1.32–2.19	2.17	1.67–2.76
≥ 10 years	27	1.73	1.15–2.47	2.63	1.76–3.76
Persons without ASD		1	1	1	1

^a^Estimates of relative risk were adjusted for calendar year, age, maternal and paternal age, sex, parental history of psychiatric illness, place of residence at time of birth and the interaction of age with sex

^b^Estimates of relative risk were adjusted for calendar year, age, maternal and paternal age, sex, parental history of psychiatric illness, first psychiatric hospital contact for any other disorder, place of residence at time of birth and the interaction of age with sex

The risk of receiving a later diagnosis of OCD did not differ between patients with in- and outpatient contacts (IRR = 3.24, 95% CI = 2.20–4.58; IRR = 2.35, 95% CI = 2.04–2.70) leading to an autism spectrum diagnosis. Additionally, a prior diagnosis of an autism spectrum disorder increased the risk for OCD with predominantly obsessional thoughts (IRR = 1.72, 95% CI = 1.24–2.32) or predominantly compulsive acts (IRR = 2.40, 95% CI = 1.79–3.16). The IRR for OCD was only increased in individuals with autism spectrum disorders without comorbid mental disabilities (IRR = 2.02; 95% CI = 1.77–2.31). Individuals with a prior diagnosis of Asperger’s syndrome, other and unspecified pervasive developmental disorders were at a significantly enhanced risk of receiving a later OCD diagnosis (see Table 2).

Longitudinal analyses showed that individuals first diagnosed with OCD had a 4-fold higher risk (IRR = 3.91, 95% CI = 3.46–4.40) of receiving a later diagnosis of an autism spectrum disorder compared with individuals without OCD during the follow-up period. The independent effect of a prior OCD diagnosis over and above the effect of a psychiatric hospital contact per se was estimated to increase the IRR of autism spectrum disorders to 4.73 (95% CI = 4.19–5.32). Excluding the first year after diagnosis, the effect of a prior OCD diagnosis on the risk to be diagnosed with an autism spectrum disorder was relatively stable overtime (see Table 4).

**Table 4:** Incidence Rate Ratio of Autism Spectrum Disorders in Persons with a Diagnosis of Obsessive-compulsive Disorder (OCD; 1994–2012).

		First Adjustment^a^		Second Adjustment^b^	
Time Since OCD Diagnosis	Cases	Incidence Rate Ratio	95% CI	Incidence Rate Ratio	95% CI
< 1 year	135	8.32	6.98–9.83	9.64	8.09–11.38
1 year	42	3.38	2.45–4.51	3.90	2.83–5.20
2 years	29	2.91	1.97–4.10	3.45	2.34–4.87
3 years	31	2.29	1.58–3.20	2.84	1.95–3.96
5 years	30	2.30	1.57–3.24	2.87	1.96–4.02
≥ 10 years	14	2.08	1.17–3.38	2.87	1.61–4.65
Persons without OCD	17893	1	1	1	1

^a^Estimates of relative risk were adjusted for calendar year, age, maternal and paternal age, sex, parental history of psychiatric illness, place of residence at time of birth and the interaction of age with sex

^b^Estimates of relative risk were adjusted for calendar year, age, maternal and paternal age, sex, parental history of psychiatric illness, first psychiatric hospital contact for any other disorder, place of residence at time of birth and the interaction of age with sex

The risk of receiving a later diagnosis of an autism spectrum disorder did not differ between patients with in- and outpatient (IRR = 5.03, 95% CI = 4.01–6.22; IRR = 4.66, 95% CI = 4.11–5.25) contacts leading to an OCD diagnosis. Additionally, the risk to be diagnosed with autism spectrum disorders was similarly increased in OCD patients with predominantly obsessional thoughts (IRR = 2.91, 95% CI = 2.01–4.04) or predominantly compulsive acts (IRR = 3.68, 95% CI = 2.62–5.01). Patients with autism spectrum disorders previously diagnosed with OCD were on average older than patients without OCD (see S2 Table). A prior hospital contact for OCD increased the IRR for autism spectrum diagnoses with (IRR = 3.10; 95% CI = 2.06–4.45) and without comorbid mental disabilities (IRR = 4.89; 95% CI = 4.67–5.13), although the IRR was significantly higher for an autism spectrum diagnosis without a comorbid diagnosis for mental disabilities. Individuals with a prior diagnosis of OCD were at an enhanced risk to be diagnosed with all types of autism spectrum disorders (see Table 1).

## Discussion

To the best of our knowledge this is the first large-sale study exploring patterns of comorbidity, longitudinal risks, and shared familial risks between autism spectrum disorders and OCD. The results of the current population-based study confirm the findings of previous reports, suggesting that OCD are far more common in individuals with autism spectrum disorders than would be expected by chance. In longitudinal analyses, we found that an initial diagnosis of autism spectrum disorders increased the risk of a later diagnosis of OCD, and vice versa. A personal history of autism spectrum disorders doubled the risk of receiving a diagnosis of OCD later in life, whereas a personal history of OCD quadrupled the risk of being diagnosed with an autism spectrum disorder later in life. Finally, the parental analyses showed considerable familial links between these disorders.

The results of our study coincide with two small-sized studies similarly reporting a link between autism spectrum disorders and OCD [27, 28]. Brimacombe et al [27] suggested that families of autistic children carried a higher burden of OCD, as the rates of OCD observed in family members exceeded the rates for the general population. Alike, Bolton et al [28] assumed that OCD might index an underlying liability to autism spectrum disorders. In their study OCD was not only more often observed in relatives of patients with autism spectrum disorders, but patients with OCD were also more likely to display autistic-like symptoms such as social and communication impairments [28].

A prior diagnosis of autism spectrum disorders in the patients and the parents increased the risk to be subsequently diagnosed with OCD. An especially enhanced risk was observed for Asperger’s syndrome, other pervasive developmental disorder and unspecified pervasive developmental. However, autism spectrum disorders with comorbid diagnosis of mental disabilities did not increase the risk of OCD. Some individuals with autism spectrum disorders have been reported to display such high levels of checking, ordering, and obsessing that a comorbid diagnosis of OCD can be justified. Gross-Isseroff et al [14] even proposed a putative autistic-compulsive syndrome for the overlap zone of the two disorders. As OCD is treatable in individuals with autism spectrum disorder [29] and the comorbidity results in high levels of distress and burden, and significant economic costs [30–33], it is important to understand how OCD manifests in this group.

Although only a small number of patients diagnosed with an autism spectrum disorder had a prior hospital contact for OCD (1.55%), a prior diagnosis of OCD in the patients and the parents increased the risk to be diagnosed with all types of autism spectrum disorder. An especially enhanced risk was observed for types of autism spectrum disorders that tend to be diagnosed a later age, which might explain the later age at diagnosis in individuals diagnosed with comorbid OCD. The results suggest that a small proportion of individuals with OCD may make a transition to autism spectrum disorders although the possibility of initial misclassification of ASD as OCD cannot be ruled out. Individuals who received an OCD diagnosis before an autism spectrum disorders diagnosis might have manifested behavioral abnormalities (e.g., repetitive behaviors) in early childhood but deficits in social interaction and social communication were not as readily apparent until a later age when social demands exceeded their capacities. OCD might further exacerbate preclinical symptoms or indicate sub-threshold autism spectrum disorders, as suggested previously [34–36].

Although, some studies report that patients with OCD and autism spectrum disorders can be distinguished on the basis of their current repetitive behaviors and thoughts [29, 37], differential diagnoses of OCD and autism spectrum disorders can be difficult to establish. Especially the stereotyped behaviors described as part of the broader autism phenotype might resemble the compulsive behaviors of patients with OCD; however, these behaviors are of different psychological quality [38]. A marked characteristic of OCD is considered to be its ego-dystonic nature, where thoughts and compulsions experienced or expressed are not consistent with the individual's self-perception and the patients try to resist them. Autistic repetitive behaviors in contrast might be pleasurable for the patients and patients might even react aggressively if they are hindered in these activities. Assessment of obsessive symptoms can be particularly difficult when the subject’s language and intelligence is compromised; accordingly some subjects cannot be truly evaluated. Despite these difficulties, the sensitivity of classification criteria for autism can be considered as remarkable high [39, 40]. In this study, patients with a prior OCD onset in contrast to other patients with autism spectrum disorders displayed an OCD typical female to male gender ratio supporting the validity of the diagnosis.

Strengths of the study are the prospective design and use of the population-based nationwide registers in Denmark. This approach enabled us to examine a large study population where all exposures were recorded independently of the outcome, minimizing probable selection or recall bias effects. The biggest limitation of the current study may be that OCD is underrepresented in the Danish national register compared with autism spectrum disorders. This is largely due to the fact, that OCD rarely requires hospitalization and is often treated by general practitioners and many sufferers do not seek help. Hence, the patterns of comorbidity, longitudinal risks, and shared familial risks of OCD and autism spectrum disorders might not be generalizable to OCD of milder severity. However, a prior OCD diagnosis assigned in in- or outpatients settings was found to increase the risk of receiving a diagnosis of autism spectrum disorders, and vice versa. Thus the observed associations are unlikely to be solely due to especially severe forms of the OCD and autism spectrum disorders requiring inpatient specialist treatment. A further limitation of our study is that individuals not yet diagnosed with an autism spectrum disorder might have displayed unspecific psychiatric symptoms resulting in possible diagnostic misclassification, which might have affected the results. Diagnostic uncertainty might explain the especially high risk rates for autism spectrum disorders and OCD within the first months after the initial diagnosis. Although a previous study reported high validity of the reported childhood autism diagnosis in the Danish Psychiatric Central Register [39], the study was based on a younger cohort of children and may not be generalizable to persons receiving an autism spectrum diagnosis in adolescence or adulthood.

The etiology of complex disorders such as autism spectrum disorders is commonly assumed to be manifold and heterogeneous. Given the substantial heritability estimates for OCD [41, 42] and autism spectrum disorder [43, 44] shared genetic liability may constitute one of a variety of pathways linking OCD to autism spectrum disorders. Interestingly, the relative risk of autism spectrum disorders in children of parents diagnosed with OCD in our study was quite similar to the relative risk of second- and third-degree relatives of OCD patients to develop OCD [42]. In addition several studies provide evidence for common environmental risk factors [42, 43] of OCD and autism spectrum disorders.

## Conclusions

The high comorbidity, sequential risk, and shared familial risks between OCD and autism spectrum disorders are suggestive of partially shared etiological mechanisms between these severe mental disorders. Probable overlaps in etiological factors of OCD and autism spectrum disorders have been consistently suggested in preclinical, neuroimaging and neurochemical studies showing that the dopaminergic, glutamatergic and serotonergic systems are implicated in the pathophysiology of both disorders [13, 45–49]. Furthermore, shared environmental risk factors such as advanced paternal age, obstetric complications and infections have been postulated [50–57]. Future research is needed to identify shared genetic and environmental risk factors of OCD and autism spectrum disorders.

## Supporting Information

S1 Table Mental Comorbidities among Individuals with Obsessive-Compulsive Disorder or Autism Spectrum Disorders (1994–2012). (DOCX)

S2 Table Age Distribution of Specific Diagnoses of Autism Spectrum Disorders in Relation to a Prior Diagnosis of Obsessive-compulsive Disorder (OCD; 1994–2012). (DOCX)
