# Supplementary material for: Obsessive-Compulsive Disorder and Autism Spectrum Disorders: Longitudinal and Offspring Risk
Source: PLoS One. 2015 Nov 11;10(11):e0141703. doi: 10.1371/journal.pone.0141703 (PMC4641696; doi:10.1371/journal.pone.0141703)
Supplement: S1 Table — (DOCX) [file pone.0141703.s001.docx]

|  |  |  |  |  |  |  |
| --- | --- | --- | --- | --- | --- | --- |
| S1 Table. Mental Comorbidities among Individuals with Obsessive-Compulsive Disorder or autism Spectrum disorders (1994-2012) | | | | | | |
|  |  |  |  |  |  |  |
|  |  |  | Obsessive-Compulsive Disorder |  | Autism Spectrum Disorders |  |
|  |  |  |  |  |  |  |
|  | Comorbid Diagnoses |  | N |  | N |  |
|  |  |  |  |  |  |  |
|  | Mental Disabilities |  | 38 |  | 3072 |  |
|  | Attention Deficit Hyperactivity Disorder |  | 906 |  | 3948 |  |
|  | Anxiety Disorders |  | 2151 |  | 665 |  |
|  | Depression |  | 2945 |  | 1568 |  |
|  |  |  |  |  |  |  |
|  |  |  |  |  |  |  |
|  |  |  |  |  |  |  |
