# Supplementary material for: Obsessive-Compulsive Disorder and Autism Spectrum Disorders: Longitudinal and Offspring Risk
Source: PLoS One. 2015 Nov 11;10(11):e0141703. doi: 10.1371/journal.pone.0141703 (PMC4641696; doi:10.1371/journal.pone.0141703)
Supplement: S2 Table — (DOCX) [file pone.0141703.s002.docx]

|  |  |  |  |  |  |  |  |  |
| --- | --- | --- | --- | --- | --- | --- | --- | --- |
| S2 Table. Age Distribution of Specific Diagnoses of Autism Spectrum Disorders in Relation to a Prior Diagnosis of Obsessive-compulsive Disorder (OCD; 1994-2012) | | | | | | | | |
|  |  |  |  |  |  |  |  |  |
|  |  |  | Autism cases in general | |  | Autism cases with prior OCD | |  |
|  |  |  |  |  |  |  |  |  |
|  | Diagnosis |  | Mean | Standard Deviation |  | Mean | Standard Deviation |  |
|  |  |  |  |  |  |  |  |  |
|  | Childhood Autism |  | 9.42 | 7.13 |  | 13.94 | 8.90 |  |
|  |  |  |  |  |  |  |  |  |
|  | Atypical Autism |  | 12.37 | 7.01 |  | 15.23 | 5.32 |  |
|  |  |  |  |  |  |  |  |  |
|  | Asperger's Syndrome |  | 14.90 | 8.56 |  | 19.08 | 9.02 |  |
|  |  |  |  |  |  |  |  |  |
|  | Other Pervasive Developmental Disorder |  | 11.09 | 5.50 |  | 14.75 | 5.88 |  |
|  |  |  |  |  |  |  |  |  |
|  | Unspecified Pervasive Developmental Disorder |  | 11.56 | 6.81 |  | 18.01 | 9.11 |  |
|  |  |  |  |  |  |  |  |  |
|  |  |  |  |  |  |  |  |  |
|  |  |  |  |  |  |  |  |  |
|  |  |  |  |  |  |  |  |  |
